# Interventions to increase adherence in patients taking immunosuppressive drugs after kidney transplantation: a systematic review of controlled trials

**DOI:** 10.1186/s13643-017-0633-1

**Published:** 2017-11-29

**Authors:** Tim Mathes, Kirsten Großpietsch, Edmund A. M. Neugebauer, Dawid Pieper

**Affiliations:** 1 Institute for Research in Operative Medicine, Witten/Herdecke University, Ostmerheimer Str. 200, Building 38, 51109 Cologne, Germany; 2 Institute for Health Economics and Clinical Epidemiology of the University of Cologne, Gleueler Str. 176-178, 50935 Cologne, Germany; 3 Brandenburg Medical School Theodor Fontane, Neuruppin, Germany

**Keywords:** Immunosuppressive drugs, Kidney transplantation, Patient adherence, Compliance, Systematic review

## Abstract

**Background:**

Immunosuppressive drugs have to be taken through the whole duration of kidney transplant survival to avoid rejection. Low adherence can increase the risk of allograft rejection.

The objective was to evaluate the effectiveness of adherence-enhancing interventions (AEI) in kidney transplantation recipients taking immunosuppressive drugs.

**Methods:**

A search was performed in Medline, Embase, CINAHL, and PsycINFO. The search was performed in May 2016. We included comparative studies on AEI for kidney transplant recipients taking immunosuppressive drugs. The primary outcome was medication adherence. All identified articles were screened according to the predefined inclusion criteria. The risk of bias was assessed with the Cochrane risk of bias tool. Study selection and risk of bias assessment were performed by two reviewers independently. Data were extracted in standardized tables. Data extraction was verified by a second reviewer. All discrepancies were resolved through discussion. Data were synthesized in a structured narrative way.

There is no registered or published protocol for this systematic review.

**Results:**

We identified 12 studies. The number of participants ranged from 24 to 1830. Nine studies included adults, two children, and one adults and children. Risk of bias was high. The main reasons for high risk of bias were inadequate allocation sequence (confounding) and that studies were not blinded.

Eleven studies evaluated AEI consisting of educational and/or behavioral components. All these studies showed an effect direction in favor of the intervention. Intervention effect was only moderate. Most adherence measures in studies on educational and behavioral interventions showed statistically significant differences. Studies that combined educational and behavioral intervention components showed larger effects. All studies that were statistically significant were multimodal. Studies that included an individualized component and more intensive interventions showed larger effects.

One study evaluated a reminder system. Effect size was not reported. This study showed no statistical significant difference (*p* > 0.05).

**Conclusion:**

Educational and behavioral AEI can increase adherence. In particular, multimodal and individualized interventions seem promising. However, because of the small effect, the high risk of bias, and the invalidity of adherence measures, the actual benefit of adherence interventions for an unselected patient population (i.e., including also adherent patients) seems limited. No conclusion is possible for interventions combining adherence-enhancing components that address intentional (behavioral) as well as unintentional adherence (reminder).

**Electronic supplementary material:**

The online version of this article (10.1186/s13643-017-0633-1) contains supplementary material, which is available to authorized users.

## Background

Chronic kidney diseases are a health problem worldwide [1]. For end-stage kidney diseases, transplantation is the first choice therapy because of higher quality of life, lower mortality, and lower costs compared to long-term dialysis [2–4]. Kidney transplantation is one of the most frequent transplantations. About 69% of all solid transplantations are kidney transplantation [5].

Immunosuppressive drugs have to be taken through the whole duration of allograft survival to avoid rejection [6]. Lifelong adherence (process by which patients take their medication as prescribed [7]) to immunosuppressive drugs is important to prevent graft failure [8, 9]. Furthermore, non-adherence can result in higher lifetime costs [10]. Studies have shown that 22–30% of patients are non-adherent, nevertheless [8, 9].

Adherence-enhancing interventions have the potential to improve health outcomes and reduce health care costs [11–13]. However, systematic reviews on adherence interventions show often heterogeneous results [11, 13].

A previous systematic review of interventions to improve adherence in adult renal transplant recipients comes to the conclusion that behavioral adherence interventions and multimodal interventions can increase adherence. We performed a systematic review on the same topic because we were aware of some new studies. Moreover, the previous systematic review included also studies without a control group and the risk of bias of included studies was not assessed which might lead to overoptimistic results. The aim of this systematic review was to assess the effectiveness of adherence-enhancing interventions in patients taking immunosuppressive drugs after kidney transplantation.

## Methods

This systematic review is reported according to the recommendations of the “Preferred Reporting Items for Systematic Reviews and Meta-Analyses” (PRISMA) statement (PRISMA-checklist see Additional file 1) [14].

### Information sources

A systematic literature search was performed in the databases Medline (via Pubmed), Embase (via Embase), CINAHL (via Ebsco), and PsycINFO (via Ebsco). We constructed an electronic search strategy c using text words and medical subject headings (MeSH terms) related to adherence, kidney transplantation, and immunosuppressive drugs (the search filters are available in Additional file 2) for each database. The search was last updated in May 2016. We did not limit the publication date and the language in the electronic search strategy. We cross-checked the references of included publications and systematic reviews on similar topics know to us.

### Study selection

We predefined the following inclusion criteria:

1. Patients taking orally immunosuppressive drugs because of kidney transplantation.
2. Intervention including a behavioral, educational or reminder component with the aim to increase patient adherence to immunosuppressive therapy.
3. Outcome/adherence measure: adherence (to immunosuppressive drugs [7].
4. Study type: randomized controlled trials (RCT), controlled clinical trials (non-randomized-trials), and cohort studies.
5. Publication language: English, German, French, or Spanish.

We only included studies on educational, behavioral, and reminder interventions. We excluded studies assessing a drug regime simplification intervention (different dosages or formulations). This decision was made because different dosages or formulations can influence the efficacy and adverse event profile of a drug. Therefore, the most relevant outcomes for a comparison of different dosages and formulations are efficacy (might be indirectly affected by adherence) and adverse events (might indirectly affect adherence). Thus, a reliable evaluation of a medication simplification adherence intervention must assess efficacy, adverse events, adherence, and their relation and consequently would require other inclusion criteria. Moreover, we assumed that in clinical practice generally, the most effective drug and dosage for each patient is scheduled. For this reason, we considered adherence interventions that can be applied for a still implemented immunosuppressive drug regime of higher clinical relevance.

In all intervention arms, the same drug regime had to be scheduled to ensure comparability of groups.

We followed the European expert panel for adherence that recommends to differentiate the analyses of adherence in initiation, implementation, and discontinuation [7]. Immunosuppressive drugs are normally initiated during hospital stay and must be taken the rest of life. Therefore we only considered adherence, while the immunosuppressive drug is implemented in this systematic review. Studies only on discontinuation and initiation measures were not included.

We screened all titles and abstracts and subsequently all full texts of titles and abstracts that appeared relevant. Two reviewers performed the title/abstract and the full-text screening independently. All discrepancies between the reviewers were discussed until a consensus was reached. The authors of the studies were contacted in case of any unclear inclusion criteria.

### Assessment of risk of bias

The Cochrane risk of bias tool was used for the risk of bias assessment of the included studies (evaluation criteria see Additional file 1) [15]. The criteria that concern outcomes were assessed for the adherence measures. The criteria were rated with “low risk of bias” (+), “high risk of bias (-),” or “unclear risk of bias” (?).

We also assessed studies without randomization (cohort studies, controlled trials) with the Cochrane risk of bias tool because we planned no quantitative synthesis and expected strong heterogeneity between studies [16].

The risk of bias assessment was performed by two reviewers independently. Any discrepancies were resolved in a discussion until a consensus.

### Data extraction and synthesis

The data were extracted in standardized beforehand piloted tables. We extracted information on study design, patient characteristics, region and setting, immunosuppressive drugs, intervention and comparator (intervention components with short descriptions, frequency of delivery, duration), the duration of intervention and duration of observation period, the measure (e.g., mean dose taken, percent adherent patients) and measurement (e.g., electronic monitoring) of adherence, and the results for adherence. Furthermore, data on results for pre-specified patient-important outcomes (mortality, health-related quality of life, graft reaction, hospital admissions, adverse events) and other adherence measures (initiation, discontinuation, persistence) were extracted for complementary analysis. Our focus was the long-term effect of the intervention (i.e., the sustainability of the intervention) because immunosuppressive drugs have to be taken lifelong. Therefore, we extracted results of the last follow-up (unless otherwise indicated). If reported in the article, we extracted means or rates per groups and confidence intervals or exact *p* values (Table 3). Otherwise, we tried to extract data as detailed as possible with the given information in the article. Data extraction was performed by one reviewer and verified by a second to ensure correctness of data and adequacy of interpretation.

Experience [17, 18] from other projects on adherence interventions showed high study heterogeneity regarding differences in the adherence interventions (different components, differences in content of components), adherence measure (e.g., mean doses taken, proportion of adherent patients), and adherence measurements (e.g., pill count, questionnaires). Therefore, a meta-analysis was not planned beforehand because a quantitative data synthesis would not have been meaningful [16]. The evidence was synthesized according to guidelines for structured narrative synthesis instead [19].

A *p* value below 0.05 for the primary outcome (two sided) was considered statistically significant.

There was no protocol published for this review. However, all study selection criteria, data designated for extraction, methods for risk of bias assessment, and the methods for evidence synthesis were specified before the conduct of the review and not changed thereafter.

The systematic review was not registered in PROSPERO.

## Results

### Literature search

**Fig. 1:**
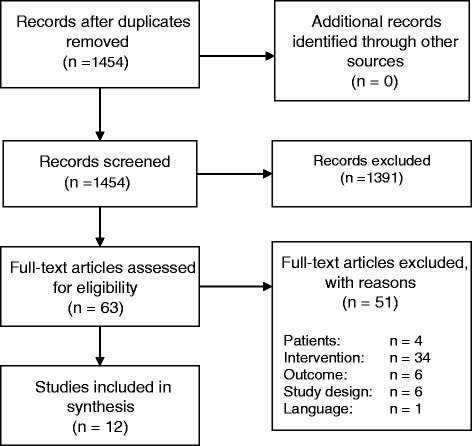
The PRISMA flow-diagram of the study selection process

The selection process is illustrated in the PRISMA flow-diagram (see Fig. 1). The literature search resulted in 1454 articles after electronic removal of duplicates (EndNote X5). We assessed 63 titles and abstracts as potentially relevant and screened the full texts in detail. We excluded 51 publications. It was not necessary to contact authors to clarify the inclusion. The cross-check of references of systematic reviews on the same topic and of the included studies revealed no further relevant articles. Twelve publications satisfied all inclusion criteria and were finally included [20–31].

### Characteristics of included studies

The description for each study is presented in Table 1.

**Table 1:** Description of studies

Author, year	Study design	Included patients/analyzed patients (I, C)	Setting and region	Patient characteristics (intervention/control or whole population)	Medication (dosing frequency)	Intervention	Control	Intervention period; observation period
Annunziato 2015	Cohort study (retrospective chart review)	Included *n* = 25 Analyzed *n* = 22 (12, 10)	Two pediatric and adult kidney transplant service; USA	Age (mean, years) 21.68/21.03 Male 42%/40% White 25%/0 African American 17/10 Hispanic 17%/70% Asian 33%/0 Other race 8%/20% Glomerular 64%/13% Non-glomerular 36%/88% Standard deviation of tacrolimus blood levels (mean) 1.98/2.42	NR	*Auspices of the transition coordinator* Identifying and addressing gaps in self-management Discussing transfer process including fears and concerns with patient and patient’s family members Solving identified problems with patient, patient’s family, and team members Facilitating last appointment in pediatrics Completing “transition checklist” during last visit in pediatrics Providing patient and patient’s family with information about their soon-to-be adult providers Sharing impressions with members of adult team	Standard care	1 year; 1 year
Breu-Dejean 2016	RCT	Included *n* = 110 (55, 55) Analyzed *n* = NR (12, 10)	Outpatient clinic at Toulouse University Hospital; Toulouse, France	Age (mean, years) 49.7/47.9 Number of immunosuppressants (mean) 2.8/2.7 Male 56.4%/49.1% Single 27.3%/34.5% Adherence score at first evaluation (mean) 29.9/32.0	Cyclosporine (dosing NR), Sirolimus (dosing NR), Tacrolimus (dosing NR), Mycophenolate mofetil (dosing NR), Enteric-coated mycophenolate sodium (dosing NR), Prednisone (dosing NR), Azathioprine, Everolimus (dosing NR)	Psychoeducational intervention (every week) Conducted by a multidisciplinary team that included 1 physician, 1 psychologist, 2 nurses, 1 kinesiotherapist, 1 dietician, and 1 social worker Main objectives: to provide information about disease and to translate this information into a form that enabled to gain increased competence during normal daily life	Standard care	8 weeks; 10 years
Chisholm 2001	RCT	Randomized *n* = 24 (12, 12) Analyzed *n* = 24 (12, 12)	Medical College of Georgia (MCG) Hospital and Clinics in Augusta, Georgia, USA	Age (mean, years) 49.2 (10.2) Male 75% Caucasian 58.3% African–American 37.5% Hispanic 4.2%	Cyclosporine (dosing NR) Tacrolimus (dosing NR)	*Clinical pharmacy services* Medication histories and reviews (monthly) Clinical pharmacist: counseling patients (verbally/written) concerning medications, recommendations to nephrologists, contact number given to patients Assessment of patient understanding of medication therapy Clinical pharmacist-patient interaction by telephone, if patient had no clinic visit within 1 month	Routine clinic services	1 year; 1 year
Chisholm 2013	RCT	Randomized *n* = 150 (76, 74) Analyzed *n* = 150 (76, 74)	Southwest USA; Avella Specialty Pharmacy	Age (mean, years) 52.78/51.32 Annual income (mean, $) 39,673.96/28,290.44 Males 56.6%/55.4% White 77.6%/82.4% African–Americans 15.8%/14.9% Hispanic 71.1%/68.9% Married 40.8%/48.6%	Cyclosporine (dosing NR) Tacrolimus (dosing NR)	Standard specialty pharmacy care + Individual behavioral adherence contracts (goal setting, motivation, social support, memory techniques, problem-solving, consequences of non-adherence) discussed with pharmacist every 3 months to discuss new goals etc.	*Standard specialty pharmacy care* Mail or telephone reminders of monthly medication refills and an adherence ‘packet’ consisting of adherence-focused educational pamphlets and a pillbox	1 year; 1 year
De Geest 2006	(Pilot) RCT	Randomized *n* = 18 (6, 12) Analyzed *n* = 13 (4, 9)	University Hospital Basel, Switzerland and Cantonal Hospital, Aarau, Switzerland	Non-adherent renal transplant recipients (identified in a previous study) Age (mean, years) 45.6 Male 78.6%	Cyclosporine, Mycophenolae-Mofetil, Tacrolimus, Sirolimus (dosing: twice daily) Azathioprin/Prednisone (dosing: once daily)	Enhanced usual care 1 home visit with assessment of reasons for adherence using EM printouts and tailored and individualized (behavioral, educational, and social support) interventions + 3 monthly telephone interviews (EM printouts for problem detection, feedback and proxy goal setting)	*Enhanced usual care* Treating physicians were informed if their patients were identified as being non-adherent or if a moderate or severe depression or suicidal ideation was suggested	3 months; 9 months
Fennell 1994	Non-randomized trial (matched according to age and sex)	Included *n* = 29 (14, 15) Analyzed *n* = NR	University of Florida; USA	Age (mean, years) 12.0 Male 59% European-Amerikan 72% African- or Latino-American 28%	NR	*Family-based program* Educational booklet with information about transplantation; Peer modeling videotape (with discussions about the need for compliance, benefits of a kidney transplant, and strategies for remembering to take medications) Medication calendar to record medication compliance Weekly rewards to the children from their parents	Usual care	NR; NR
Garcia 2015	RCT	Included *n* = 111 (55, 56) Analyzed *n* = 111 (55, 56)	Universidade Estadual Paulista; Botucatu; Brasil	Age (mean, years) 46.0/49.3 Male 56.4%/62.5%	Tacrolimus (dosing NR), Cyclosporine (dosing NR), Mycophenolate (dosing NR), Azathioprine (dosing NR), Prednisone (dosing NR)	Usual care Education/counseling sessions aimed at improving delivered by a single healthcare professional with expertise in renal transplantation (10 weekly sessions, 30 min each); diverse topics which included information about the importance of taking immunosuppressive drugs even when the graft function is normal, using a non-judgmental approach to discussing adherence and tools to integrate medication intake with the patient’s daily routine	Usual transplant patient education by the medical team regarding the immunosuppressant drugs in their first outpatient assessment after discharge	3 months; 1 year
Hardstaff 2003	RCT	Randomized *n* = 75 Analyzed at first outpatient visit: *n* = 48 (23, 25) Analyzed at period after feedback: *n* = 40 (20, 20)	NR	Stable (>1 year post-transplant) renal transplant patients	Prednisolone/Azathioprine (dosing: once daily)	Feedback about self-medication behavior at first outpatient clinic visit	Usual care	Unique at first outpatient visit (2–6 months); 4–12 months (depending on first outpatient visit)
Henriksson 2016	RCT	Included *n* = 80 (40, 40) Analyzed *n* = 80 (40, 40)	Karolinska University Hospital; Stockholm, Sweden	Age (mean) 44.3/45.0 Male 27/25	Tacrolimus (dosing: twice daily, or in “slow release” form once daily), cyclosporine (dosing: twice daily)	*Electronic medication dispenser (EMD)* Loaded with a week’s worth of medication at a time At the prescribed time for taking the medication visual and audible signals After signals this (or after the medication was taken), the EMD sent an SMS message to the web-based software, thus providing information about patient compliance Provider reviewed medication history	Standard care	2 years; 1 year
Joost 2014	Non-concurrent cohort study	Included *n* = 74 (39,35) Analyzed *n* = 67 (35, 32)	Erlangen University Hospital, Germany	Age (mean, years) 51/54 Male 77%/62 Married 83%/82%	Tacrolimus/Cyclosporin/Mycophenolic acid (dosing: twice daily)	*Intensified Care Group* Standard care + pharmaceutical care: ≥ 3 counseling sessions including educational, behavioral and technical interventions (during week 1–2), further counseling sessions during follow-up visits throughout the 12 months (≥ 1 quarterly; ≤ 1 monthly), encouraged to contact the pharmacist via phone or email	*Standard Care Group* Handout explaining post-transplant medication + 1–2 individual standardized training sessions (during 1–2 week) + scheduled follow-up visits	12 months; standard care 2 weeks
Russel 2011	(Pilot) RCT	Randomization *n* = 15 (8, 7) Analyzed *n* = 13 (8, 5)	Tertiary care transplant centre; Midwestern USA	Medication non-adherent (taking < 85% of doses before inclusion) Age (mean, years) 12.1/15.7 Male 50%/43% Caucasian 100%/57% Education level (some high school/high school) 63%/14% Married 75%/43% Pillbox use 88%/29%	≥ 1 immunosuppressive medication (medication not specified; dosing: twice daily)	*Continuous self-improvement intervention* Identification of life routines, important people, and possible solutions to enhance medication taking Individual monthly medication taking feedback Focus on changing the systems in which the person lives using the plan-do-check-act process	*Attention control intervention* Monthly educational brochures Telephone calls to review the information and to ask participants whether they have any questions about the information	6 months; 6 months (plus prior 3 months adherence screening phase)
Tschida 2013	Cohort study (retrospective claims analysis)	Before propensity score matching *n* = 1830 Propensity-matched sample *n* = 1038 (519 pairs)	Mandatory program for the commercial employers of UnitedHealthcare, USA	UnitedHealthcare enrollees receiving pharmacy and medical benefits through UnitedHealthcare	≥ 1 prescriptions for an oral transplant study drug (dosing NR)	*Specialty pharmacy program* Extensive patient education materials Monthly proactive adherence program: refill reminder, adherence screening, and if non-adherent interventions with members and physicians Transplant clinical management program: telephonic patient education, assessment of clinical status, pharmaceutical care intervention Contact number 24 h available	No intervention	1 year; 1 year after index date (the first immunosuppressive drug prescription fill date)

*NR* not reported, *RCT* randomized controlled trial

Eight of the included studies were randomized controlled trials [21–28], two retrospective cohort studies [20, 31], one a non-concurrent cohort study [30], and one a non-randomized trial [29]. In four studies, the sample size was less than 30 [24, 26, 28, 29]. One study was carried out in a clinical pharmacy [25], while the other studies were performed in hospitals. Half of the studies were performed in the USA [20, 24, 25, 28, 29, 31]. Adult patients (mean age range: 45 to 53 years) were included in nine studies. Two studies included children and adolescents [28, 29], whereby Russell et al. [28] included only youth with low baseline adherence. In one study, children as well as adults were included [20]. In the studies that were performed in the USA, most of the included patients were Caucasians [24, 25, 28, 29]. The primary prescribed immunosuppressive regimes were cyclosporine/tacrolimus, cyclosporine/tacrolimus/mycophenolic acid, and azathioprine/prednisone. Most adherence interventions consist of educational and/or (combinations) behavioral components. This implies that most studies targeted only intentional non-adherent patients. In one study, a reminder system without another measure to increase adherence was used, means primarily unintentional adherence was addressed [23]. The most often used behavioral intervention was adherence feedback to the intake behavior [26–28, 31]. Five studies compared the adherence intervention against another less intensive adherence intervention [22, 25, 26, 28, 30]. The other studies compared the adherence intervention to usual care. The duration of the intervention ranged from 8 weeks [21] to 1 year [23–25, 27, 31].Only three studies had a follow-up, i.e., the observation period was longer than the intervention period [21, 22, 26].

The applied adherence measures and measurement methods were very heterogeneous. Two studies used only tacrolimus blood levels to determine medication adherence [20, 23]. Breu-Dejean et al. [21] used of a self-developed questionnaire [21]. Chisholm-Burns et al. assessed adherence as doses taken with prescription refill [25]. In the studies by de Geest et al. [26] and Hardstaff et al. [27], patients taking a predefined dose were measured by electronic monitoring. Russell et al. [28] constructed an adherence score that allows to assess the correct timing of medication. In the study by Joost et al. [30], different adherence measures (patients taking ≥ 80 of doses, mean adherence, mean daily adherence, timing adherence, drug holidays) and measurements (electronic monitoring, prescription refill, questionnaire/self-report) were used.

The other studies applied several different measures or measurement methods. Chisholm et al. [24] used doses taken, patients taking ≥ 80% of doses and blood level concentrations. Adherence was measured by prescription refill, serum concentrations respectively. In the study by Fennell et al. [29], doses taken (pill count) and blood level concentrations were used to measure adherence. Tschida et al. [31] defined adherence as doses taken and as medication gaps. Both adherence measures were gathered with prescription refill. Also, Garcia et al. [22] performed two different measurements: mean tacrolimus blood levels and the immunosuppressant Therapy Adherence Scale.

Five studies also assessed patient-important outcomes [20–23, 30].

### Risk of bias assessment

The results of the risk of bias assessment are presented in Table 2. The overall risk of bias of the included studies was high. Most risk of bias items were rated with unclear. Because of the obvious nature of adherence-enhancing interventions, blinding of patients and intervention delivering health care professionals are not possible. Therefore, the risk of bias criteria for blinding were rated with “high risk of bias” throughout. Studies were either not randomized, and the randomized studies were small. Consequently, bias due to unmeasured confounding could not be excluded (e.g., unbalance of psychological determinates). Therefore, the item “other source of bias” was assessed as “unclear” for all studies. We could not find a “protocol for any study” and thus rated also “selective reporting” as unclear in each study. The remaining items (sequence generation, allocation concealment, incomplete outcome data) were either judge with “no” or “unclear” for at least 6 of the 12 included studies.

**Table 2:** Results of the risk of bias assessment

Author, year	Sequence generation	Allocation concealment	Blinding of participants, personnel and outcome assessors	Incomplete outcome data	Selective outcome reporting	Other sources of bias
Annunziato 2015	-	-	-	?	?	?
Breu-Dejean 2016	+	+	-	?	?	?
Chisholm 2001	?	?	-	?	?	?
Chisholm 2013	+	?	-	+	?	?
De Geest 2006	+	+	-	+	?	?
Fennell 1994	?	?	-	?	?	?
Garcia 2015	+	+	-	?	?	?
Henriksson 2016	?	+	-	?	?	?
Hardstaff 2003	?	?	-	-	?	?
Joost 2014	-	-	-	?	?	?
Russel 2011	+	+	-	+	?	?
Tschida 2013	-	-	-	?	?	?

*+* low risk of bias, - high risk of bias, *?* unclear risk of bias

### Effect of intervention

Results of individual studies are presented in Table 3.

**Table 3:** Results of included studies

Study	Adherence measure	Implementation adherence measurement (measurement time points)	Results for adherence^a^ (I vs. C; *p* or 95%CI)	Clinical and other adherence outcomes	Results for other outcomes^a^ (I vs. C; *p* or 95%CI)
Annunziato 2015	Standard deviation of tacrolimus blood levels (mean)	NR (median 8 measures)	2.68 vs. 3.37; *p* = 0.32^b^	Episodes of rejection (*n*)	3 vs.4; *p* > 0.05
Breu-Dejean 2016	NR	Questionnaire (5 months)	74.5 vs. 47.3%; *p* < 0.05 (5 months); *p* = NR (10 years)	Mortality	12.7 vs.9.1%; *p* = 0. 35
	–	–	–	Allograft survival	69.1 vs.87.3%; *p* = 0.06
Chisholm 2001	Percent of patients taking ≥ 80% (monthly)	Prescription refill (monthly at each visit)	I > C; *p* < 0.05 for 6 of 12 months (months 6–8 and 10–12); *p* > 0.05 for 6 of 12 months (1–5 and 9)	Duration of compliance since transplant	75 vs. 33.3%; *p* < 0.05
	Mean doses taken	Prescription refill (monthly at each visit)	96.1 vs. 81.6%; *p* < 0.001	Time to first non-compliance (month)	11; 95%CI 10–12 vs. 9; 95%CI 7–11
	Blood level within range (cyclosporine 250–400 ng/mL and tacrolimus 8–15 ng/mL)	Serum concentrations (monthly at each visit)	64 vs. 48%; *p* < 0.05 (whole study period)	–	–
Chisholm 2013	Mean doses taken	Prescription refill (baseline + every 3 months)	89 vs. 79%; *p* < 0.01	–	–
De Geest 2006	Patients taking < 98% and/or ≥ 1 drug holiday (no medication intake > 36 h for a twice daily dosing regimen, or > 60 h for a once daily dosing regimen)	EM (daily measurement over 9 months)	Decrease: I > C; *p* = 0.31 (3 month); *p* = 0.58 (9 months)	–	–
Fennell 1994	Mean doses taken	Pill count (4–6 weeks and 8–12 weeks)	Increase: 67% (azathioprine); 56% (prednisone) vs. 33% (azathioprine); 35% (prednisone); *p* ^b^ < 0.05% (azathioprine); *p* ^b^ < 0.02 (prednisone)	–	–
	Blood level within range (cyclosporine)	NR (4–6 weeks and 8–12 weeks)	I > C; *p* = 0.06 ^b^ (4–6 weeks); NR; ns (8–12 weeks)	–	–
Garcia 2015	Non-adherent patients (questionnaire score < 12)	Questionnaire (immunosuppressant Therapy Adherence Scale, score 0–12, 3 months)	14.5 vs. 46.4%; *p* = 0.001	Mortality	1.8 vs. 1.8%; *p* = 1.00
	Mean sum score values	Questionnaire (immunosuppressant Therapy Adherence Scale, score 0–12, 3 months)	11.82 vs.11.2; *p* = 0.001	Acute reaction	16.4 vs. 14.3%; *p* = 0.76
	Mean tacrolimus blood levels (ng/dL)	NR (at each outpatient visit up to the 3 months and were also assessed at 6 months and 1 year)	8.74 vs. 8.78; *p* = 0.93	Graft lose	1.8 vs. 7.1%; *p* = 0.17
Hardstaff 2003	Number of patients showing adherence improvement	EM (first outpatient visit and after feedback, 3 months)	6 vs. 5; NR (first outpatient visit) 6 vs. 2; NR (post intervention)	–	–
Henriksson 2016	Tacrolimus blood levels (mean)	NR (twice weekly over the first 3 months, once a week from then until 6 months, and once a month from 6 months to 1 year)	NR; *p* > 0.05	Emergency hospital admissions	22 vs. 31; *p* = 0.854
	–	–	–	Rejection	6 vs. 27; *p* = 0.054
Joost 2014	Daily adherence (Percentage of days with correct dosing)	EM (daily measurement)	91 vs. 75%; *p* = 0.014	Physical sum scale (SF-36)	I > C; *p* = 0.329
	Patients with daily adherence ≥80%		84 vs. 57%; *p* = 0.015		
	Mean doses taken		95 vs. 82%; *p* = 0.005	Mental sum scale (SF-36)	I > C; *p* = 0.419
	Patients taking ≥90% ≤ 110% of doses		84 vs. 57%; p = 0.015		
	Timing adherence (percentage doses taken within 6-h interval ± 3 h)		95 vs. 94%; *p* = 0.142	Anxiety scale (SF-36)	4.74 vs. 4.33; *p* = 0.266
	Timing adherence ≥ 80%		97 vs. 86%; *p* = 0.110		
	Patients without drug holidays (no intake > 48 h)		81 vs. 43%; *p* = 0.001	Depression scale (SF-36)	2.89 vs. 2.64; *p* = 0.193
	Mean doses taken	Pill count	97 vs. 90%; *p* = 0.008		
	Patients taking ≥ 90 ≤ 110% of doses		84 vs. 63%; *p* = 0.047		
	No missed intake during the last 2 weeks	Self report (12 months)	72 vs. 77%; *p* = 0.193		
	Fully adherent	Morisky questionnaire (12 months)	63 vs. 63%; *p* = 0.695		
Russel 2011	Mean medication adherence score (0.5 if medication-dose was taken within 3 h of prescribed 12 h time frame; 0.25 if not within 3 h but 12 h; and 0 if not within 12 h)	EM (daily measurement over 6 months)	0.88 vs. 0.77; *p* = 0.04	–	–
Tschida 2013	Mean doses taken	Prescription refill	87 vs. 83%; *p* < 0.0001	Discontinuation (≥ 60 days without medications never followed by re-initiation within the study period)	39 vs. 104; *p* < 0.0001
	Number of medication gaps (≥ 60 days without medication in post-period but followed by re-initiation before end of post-period)		29 vs. 53; *p* = 0.006		

*C* control group, *CI* confidence interval, *EM* electronic monitoring, *I* intervention group, *ns* not significant, *NR* not reported

^a^Unless otherwise indicated, all values are group means of the last follow-up

^b^ *p* value for treatment by time interaction

All studies in adults that evaluated adherence interventions including educational and/or behavioral components showed an effect direction in favor of the intervention for at least one adherence measure [22, 24, 29–31]. Most comparisons of adherence outcomes were statistically significant in these studies. However, adherence was mostly only slightly increased. In the studies that used different adherence outcomes (measures and/or measurement method), there were no conflicting effect directions. Studies in adults that combined education and a, behavioral component showed larger effects compared to studies with only one component. All studies that showed statistical significant differences were on multimodal interventions [21, 22, 24, 25, 29–31]. Larger effects were observed in studies that also included an individualized component (e.g., feedback on individual adherence behavior, discussion of individual adherence barriers) and/or that included more intensive interventions (e.g., more sessions, longer intervention period) [21, 22, 24, 25, 29–31] compared to studies without individualization and/or combination of intervention components [20, 23, 26–28].

The study that evaluated a reminder system as main component showed no statistical significant difference between groups (effect size not reported) [23].

The two studies on children (educational booklet combined with counseling video and feedback versus usual care; continuous self-improvement intervention versus education) showed a statistical significant difference [28, 29].

Patient-important outcomes showed an effect direction in favor of the adherence intervention in the two studies on an educational plus behavioral intervention component and in the study on the reminder system [22, 23, 30]. Again in the study by Breu-Dejean et al. [21], mortality was higher and graft survival was lower in the psychoeducational intervention group which was conflicting with the effect direction of the adherence measures.

## Discussion

All included studies including an educational (e.g., information on the importance of adherence) and/or behavioral intervention (e.g., feedback on the intake behavior) component showed a positive effect on adherence. Multimodal (education and behavioral) interventions showed stronger effects. One study evaluated a reminder system [23]. This study showed no statistical significant difference in adherence between groups. The confidence in the results for adherence outcomes is limited by the high risk of bias.

Individual patient characteristics and causes (intentional vs. unintentional) for non-adherence require different intervention components. A multimodal intervention increases the probability that the individual patient receives a “proper” intervention component. This is probably the reason for the observation that multimodal interventions were more effective in our systematic review. Also, other research has shown that tailoring the intervention to the patient is one of the most important factors for the success of an adherence intervention [32].

In multimodal adherence interventions, only education and behavioral components were used together. Both components primarily address intentional non-adherence (conscious decisions not to take medication). No study was identified that assessed combinations with a reminder system that address non-intentional adherence (forgetting). Consequently, information is missing on the effectiveness of combinations and synergies between reminder systems and other components or combinations of all components. In the study on the reminder system, fewer hospital admissions were recognized in the intervention group indicating also a positive effect of intervention components targeting unintentional adherence [23]. Both causes for non-adherence are common. Therefore, it can be assumed that when using only one component, either the unintentional or the intentional non-adherent population is not reached properly by the intervention [33].

Effect differences between groups were often small. One reason might be that the control group often encompasses also an adherence-enhancing component [22, 24–26, 28, 30]. In the other studies, usual care was not further explained [20, 21, 23, 27, 29, 31]. Also, in these studies, adherence-enhancing measures might be offered to the control group. Moreover, it can be assumed that a large proportion of patients with high baseline adherence were included in the study populations because low baseline adherence or high risk for non-adherence (e.g., risk groups) were not defined as inclusion criteria in most studies. Thus, there is less potential to further increase adherence (ceiling effect) and consequently also less potential to show large difference between study groups [34]. The problem of small differences between groups might be further triggered by contamination which is a known problem in educational/counseling interventions [35, 36].

The baseline adherence is only considered in three studies [25, 26, 28]. Chisholm-Burns et al. [25] adjusted the analysis for baseline adherence. Russell et al. [28] included only non-adherent patients. Both studies showed a statistical significant effect of the intervention. Even the study by Russell et al. [28] was statistically significant despite the fact that the sample size was very low. De Geest et al. [26] also included only non-adherent patients. Nevertheless, they could not prove a statistical significant difference. The cause is probably the hard definition of adherence (> 98% taking) which is very difficult to reach. Furthermore, the analysis was not adjusted for baseline adherence.

The applied adherence measures and measurement methods varied widely. Most of the applied measurement methods cannot be considered valid. Using questionnaires, pill-counts/ prescription refill tends to overestimate adherence [37, 38]. Therefore, a more valid adherence measure is the medication event monitoring system. But also electronic monitoring can lead to incorrect measurement because only the openings can be measured. Doses taken was the most frequently applied adherence measure. A drawback of the overall mean is that a quantification of patients that are sufficiently adherent to reach immunosuppression is not possible. Taking this into account, the proportion of patients reaching a specified adherence level should be chosen as the primary measure. However, this is only valid if the lower threshold of adherence that is required an effect on a patient important is determined beforehand (validated surrogate). Pharmacogenetics and pharmacodynamics and consequently the required adherence might vary from patient to patient. So, such a threshold should be selected to that effect that the proportion of patients not experience a reaction are maximized. None of the studies used such a proven adherence threshold. Moreover, most studies had no follow-up after end of intervention, which permits the assessment of sustainability of the adherence interventions.

In view of the small effect sizes, the high risk of bias, the invalidity of the adherence measures/measurement methods, and the questionable sustainability of interventions effects, a patient-important benefit seems doubtful. This problem becomes obvious in the study by Breu-Dejean et al. [21]. In this study, adherence was much higher in the intervention group; however, patient-important outcomes were conflicting.

A previous systematic review on adherence interventions in kidney transplant recipients also indicates that adherence interventions might be effective [39]. The authors concluded that behavioral interventions or a combination of behavioral, educational, and emotional components are effective in increasing adherence. On the one hand, our findings are in accordance with the conclusion that the combination of components leads to higher effectiveness. On the other, we could not prove the finding that especially behavioral interventions are promising nor identify certain promising intervention combinations. Moreover, because of the restrictions mentioned above, we consider the benefit of adherence-enhancing interventions for kidney transplant patients more skeptical in general. The reason for the different conclusions might be that we identified more controlled studies and excluded all studies without a control group. In contrast to the previous systematic review, we included studies on adolescence and excluded studies on different regimes (e.g., once daily intake versus twice daily intake). Therefore, our systematic review is based on a different body of evidence (overlap of included 50%, i.e., six studies are included in both reviews). Moreover, we assessed the risk of bias and considered the validity of adherence measures, i.e., put a greater intention on the validity of the data.

The applicability of the findings of this systematic review may be limited in other settings. Here, two aspects should be particularly mentioned. A multimodal intervention often requires a multidisciplinary team. These might not be feasible in outpatient care or small hospitals. Another aspect concerns computer-based electronic devices (e.g., electronic monitoring). Such interventions require a pre-existing IT-infrastructure and are costly. This can be a problem especially in resource-limited settings.

The presented systematic review has some methodological limitations. Firstly, a comprehensive search for gray literature was not carried out. Secondly, our evidence synthesis is partly based on a dichotomous classification of statistical significance because exact *p* values, because effect sizes or confidence limits were often not reported in the included publications.

## Conclusion

This systematic review shows that adherence interventions for kidney-transplanted recipients can increase adherence. Interventions were heterogeneous. Therefore, a definitive recommendation on a certain adherence intervention which should be implemented in clinical practice is not possible but only for ingredients that are important for an effective adherence intervention. In particular, multimodal (e.g., feedback with education) and individualized (e.g., tailored educational material) interventions seem promising. Furthermore, the intensity of intervention appears to be an important factor for the success of education and behavioral interventions. Effect sizes were small in most studies, the studies showed high risk of bias, adherence measures/measurements can be considered invalid, and most studies did not assess patient-important outcomes. Therefore, the actual benefit of adherence interventions targeting an unselected patient population (i.e., including also adherent patients) seems limited in general. Only one study evaluated a reminder system. In this study, effect sizes and statistical significance for adherence outcomes were not reported. Thus, a judgment for interventions addressing unintentional adherence is not possible. Furthermore, no study was identified that combines intervention components addressing intentional and also unintentional adherence (e.g., reminder and adherence feedback). Consequently, a conclusion for adherence interventions addressing both causes for non-adherence is also not possible. This combination seems very promising because it targets different patient populations and would therefore probably reach more patients and consequently increase the overall effectiveness.

Further, high-quality RCTs on multimodal interventions and individualized interventions (cause for non-adherence [intentional, unintentional], life situation) should be performed. The RCTs should evaluate patient-important outcomes or use an adherence measure with proven relevance in combination with a valid adherence measurement method. Furthermore, studies should focus on patients with proven non-adherence or at high risk for non-adherence (e.g., risk groups).

## Additional files

Additional file 1: PRISMA 2009 Checklist. (DOC 63 kb)

Additional file 2: Search strategies. (DOCX 15 kb)
