# Supplementary material for: Interventions to increase adherence in patients taking immunosuppressive drugs after kidney transplantation: a systematic review of controlled trials
Source: Syst Rev. 2017 Nov 29;6:236. doi: 10.1186/s13643-017-0633-1 (PMC5707897; doi:10.1186/s13643-017-0633-1)
Supplement: Supplementary file 2 — Search strategies. (DOCX 15 kb) [file 13643_2017_633_MOESM2_ESM.docx]

Additional file 2: search strategies

| **Database (provider)** | **Search strategy** |
| --- | --- |
| MEDLINE (via Pubmed) | (((transplant* [Title/Abstract] OR Tx [Title/Abstract]) AND (renal [Title/Abstract] OR kidney [Title/Abstract] OR nephrol* [Title/Abstract] OR kidney [MeSH Terms] OR nephrology [MeSH Terms])) OR RTX [Title/Abstract] OR kidney transplantation [MeSH Terms] OR kidney/ transplantation [MeSH Terms]) AND (Adherence [Title/Abstract] OR adherent [Title/Abstract] OR adhere [Title/Abstract] OR nonadherence [Title/Abstract] OR nonadherent [Title/Abstract] OR Compliance [Title/Abstract] OR “patient compliance” [MeSH Terms] OR compliant [Title/Abstract] OR comply [Title/Abstract] OR noncompliance [Title/Abstract] OR noncompliant [Title/Abstract]) AND (oral [Title/Abstract] OR administration [Title/Abstract] OR taking [Title/Abstract] OR intake [Title/Abstract] OR qd [Title/Abstract] OR “q.d.” [title/abstract] OR BID [Title/Abstract] OR “bis in die” [Title/Abstract] OR “b.i.d.” [title/abstract] OR tid [Title/Abstract] OR “t.i.d.” [title/abstract] OR Immunosuppress* [Title/Abstract] OR immunsuppress* [Title/Abstract] OR “immunosuppressive agents” [MeSH Terms] OR immunosuppression [MeSH Terms] OR “drug therapy” [MeSH Terms] OR Pharmacotherap* [Title/Abstract] OR Drug [Title/Abstract] OR Drugs [Title/Abstract] OR pill* [Title/Abstract] OR medicament* [Title/Abstract] OR medication* [Title/Abstract] OR medicin* [Title/Abstract] OR pharmaceutical* [Title/Abstract] OR tablet* [Title/Abstract]) |
| Embase (via Emabse) | (((transplant*:ti,ab OR Tx:ti,ab) AND (renal:ti,ab OR kidney:ti,ab OR nephrol*:ti,ab OR kidney/exp OR nephrology/exp)) OR RTX:ti,ab OR ‘kidney transplantation’/exp) AND (Adherence:ti,ab OR adherent:ti,ab OR adhere:ti,ab OR nonadherence:ti,ab OR nonadherent:ti,ab OR Compliance:ti,ab OR ‘patient compliance’/exp OR compliant:ti,ab OR comply:ti,ab OR noncompliance:ti,ab OR noncompliant:ti,ab) AND (oral:ti,ab OR administration:ti,ab OR taking:ti,ab OR intake:ti,ab OR ‘quaque die’:ti,ab OR ‘qd’:ti,ab OR ‘q.d.’:ti,ab OR ‘bid’:ti,ab OR ‘bis in die’:ti,ab OR ‘b.i.d.’:ti,ab OR ‘ter in die’:ti,ab OR ‘tid’:ti,ab OR ‘t.i.d.’:ti,ab OR Immunosuppress*:ti,ab OR immunsuppress*:ti,ab OR ‘immunosuppressive agent’/exp OR ‘immunosuppressive treatment’/exp OR ‘drug therapy’/exp OR Pharmacotherap*:ti,ab OR Drug:ti,ab OR Drugs:ti,ab OR pill*:ti,ab OR medicament*:ti,ab OR medication*:ti,ab OR medicin*:ti,ab OR pharmaceutical*:ti,ab OR tablet*:ti,ab) AND article/it AND [embase]/lim |
| CINAHL (via Ebsco) | (MH „Kidney Transplantation+“ OR AB RTX OR TI RTX OR ((TI transplant* OR AB transplant* OR TI Tx OR AB Tx) AND (MH „Kidney+“ OR MH „Nephrology“ OR AB renal OR TI renal OR TI kidney OR AB kidney OR TI nephrol* OR AB nephrol*))) AND (MH „Patient Compliance+“ OR MH „Medication Compliance+“ OR MH „Adherence Behaviour (Iowa NOC)“ OR TI adherence OR AB adherence OR AB adherent OR TI adherent OR TI adhere OR AB adhere OR AB nonadherence OR TI nonadherence OR TI nonadherent OR AB nonadherent OR AB compliance OR TI compliance OR TI compliant OR AB compliant OR AB comply OR TI comply OR TI noncompliance OR AB noncompliance OR AB noncompliant OR TI noncompliant) AND (MH „Immunosuppressive Agents+“ OR MH „Immunosuppression+“ OR MH „Drug Therapie+“ OR TI oral OR AB oral OR AB administration OR TI administration OR TI taking OR AB taking OR AB intake OR TI intake OR TI qd OR AB qd OR AB q.d. OR TI q.d. OR AB BID OR TI BID OR TI b.i.d. OR AB b.i.d. OR AB tid OR TI tid OR TI t.i.d. OR AB t.i.d. OR AB immunosuppress* OR TI immunosuppress* OR AB immunsuppress* OR AB pharmacotherap* OR TI pharmacotherap* OR TI drug OR AB drug OR AB drugs OR TI drugs OR TI pill* OR AB pill* OR AB medicament* OR TI medicament* OR TI medication* OR AB medication* OR AB medicin* OR TI medicin* OR TI pharmaceutical* OR AB pharmaceutical* OR AB tablet* OR TI tablet*) |
| PsycINFO ((via Ebsco) | (((DE „organtransplantation“ OR TI transplant* OR AB transplant* OR TI Tx OR AB Tx) AND (AB renal OR TI renal OR TI kidney OR AB kidney OR DE kidneys OR DE kidney diseases OR TI nephrol* OR AB nephrol*)) OR AB RTX OR TI RTX) AND (TI adherence OR AB adherence OR AB adherent OR TI adherent OR TI adhere OR AB adhere OR AB nonadherence OR TI nonadherence OR TI nonadherent OR AB nonadherent OR AB compliance OR TI compliance OR DE compliance OR DE treatment compliance OR TI compliant OR AB compliant OR AB comply OR TI comply OR TI noncompliance OR AB noncompliance OR AB noncompliant OR TI noncompliant) AND (TI oral OR AB oral OR AB administration OR TI administration OR TI taking OR AB taking OR AB intake OR TI intake OR TI qd OR AB qd OR AB q.d. OR TI q.d. OR TI quaque die OR AB quaque die OR AB BID OR TI BID OR TI b.i.d. OR AB b.i.d. OR AB bis in die OR TI bis in die OR TI ter in die OR AB ter in die OR AB tid OR TI tid OR TI t.i.d. OR AB t.i.d. OR AB immunosuppress* OR TI immunosuppress* OR TI immunsuppress* OR AB immunsuppress* OR AB pharmacotherap* OR TI pharmacotherap* OR TI drug OR AB drug OR AB drugs OR TI drugs OR TI pill* OR AB pill* OR AB medicament* OR TI medicament* OR TI medication* OR AB medication* OR AB medicin* OR TI medicin* OR TI pharmaceutical* OR AB pharmaceutical* OR AB tablet* OR TI tablet* OR DE immunodepression OR DE drug therapy) |
